# Quantitative assessment of redness in Asian patients with recurrent blepharitis: the utility of cross-polarized light

**DOI:** 10.3389/fmed.2025.1594764

**Published:** 2025-04-30

**Authors:** Yue Tan, Wenjia Sun, Yue Yin

**Affiliations:** ^1^Eye & ENT Hospital, Fudan University, Shanghai, China; ^2^NHC Key Laboratory of Myopia and Related Eye Diseases, Chinese Academy of Medical Sciences, Shanghai, China; ^3^Dermatology Department, Shanghai United Family Hospital, Shanghai, China

**Keywords:** blepharitis, cross-polarized light, redness area proportion, telangiectasis, hyperemia

## Abstract

**Purpose:**

To establish an objective and quantitative method of evaluation redness in patients with blepharitis.

**Methods:**

12 adult Asian patients with recurrent blepharitis were enrolled in the case group. 24 healthy controls, matched for age and gender in a 1:2 ratio, were recruited as the health group. Population characteristics, related medical histories and clinical indices of both groups were recorded. Redness area proportion of eyelid (RAE%) and eyelid margin (RAM%), were evaluated by cross-polarized light (Vplus^®^) and image processing. Samples of eyelid margin secretions were collected for proteomics.

**Results:**

The population characteristics and clinical indices of two groups adhered to the study design. The score chart of principal component analysis shows significant differences in protein expression of eyelid secretions between two groups. The mean ± SD (standard deviation) values of RAE% and RAM% in the health group were 1.88 ± 2.53% and 1.63 ± 2.04%, respectively. The case group had the RAE% of 6.54 ± 7.20% (mean ± SD) and the RAM% of 17.14 ± 18.90% (mean ± SD), which were both significantly higher than those in the health group (all *P* < 0.05). Within the case group, RAM% was significantly higher than RAE% (*P* = 0.019), which means the redness in case group being concentrated within eyelid margin rather than the whole eyelid. RAM% had higher positive correlation coefficients with cornea staining, meibum quality, and meibomian gland dropout compared to RAE%. And RAM% was also positively associated with more protein expression levels in eyelid margin secretions.

**Conclusion:**

By using cross-polarized light, characteristic changes of redness can be observed in patients with recurrent blepharitis. RAM%, has a great potential value for standardizing and quantifying the inflammatory status of blepharitis.

## Introduction

Blepharitis is a common inflammatory disease of the eyelid margin, typically presenting as a chronic disorder (1, 2). The eyelid margin serves as the junction between the skin and mucous membrane, where the eyelashes and the orifices of the meibomian gland are located. During blinking, the eyelid margin functions to distribute tears, resulting in repeated contact with the cornea and bulbar conjunctiva (3). Inflammation of the eyelid margin can result in a range of comorbidities in adjacent tissues, such as keratitis, conjunctivitis, meibomian gland dysfunction, chalazion, hordeolum and trichiasis (1, 4, 5). Due to the easier detection of signs and symptoms associated with these related diseases, blepharitis is often overlooked clinically, leading to misdiagnosis (e.g., blepharitiskeratoconjunctivitis, BKC) (6), which has been deemed blepharitis a “diagnostic and therapeutic enigma” (1). Furthermore, blepharitis has been reported having a high recurrence rate as high as 50% (7). Not few patients suffer from misdiagnose and relapses, potentially even affecting visual acuity (6, 8). The difficulty in diagnosing blepharitis is also associated with existing diagnostic standard, which primarily rely on the history and clinical exam (7, 9). In other words, it also relies on the clinician’s experience. The current lack of objective assessment for blepharitis reduces comparability between studies and limits comparative research and development in this area.

Redness is one of the characteristic manifestations of inflammation (10). Under the influence of inflammatory mediators, such as complement C5 (11, 12), body tissues undergo vasodilation and increased blood flow (13). Therefore, assessing redness in inflammatory diseases is essential for both diagnosing and determining the severity of the condition. Previous studies on blepharitis and related diseases, such as meibomian gland dysfunction (MGD), have frequently included the severity of redness as an outcome (7, 14). However, the evaluation results were mostly based on subjective scoring by researchers (6, 14). In dermatology, meanwhile, objective methods are well-established for chronic inflammatory diseases, such as acne or rosacea (15–17). Dermatologists use cross-polarized light imaging to capture dermal hyperemia and telangiectasia (15–17), resulting in highly repeatability and comparable redness evaluations due to standardized imaging conditions and systematic data processing. The utility of cross-polarized light effectively minimizes non-specific reflections from the skin surface, enhancing the visibility of the dermal signal. Hemoglobin is well-visualized with high quality using this lighting system (18), which makes the hyperemia and telangiectasia much more observable.

Given the inflammatory nature and affected region of blepharitis, we hypothesize that this technology has significant potential for standardizing and quantitatively assessing redness in blepharitis patients along with a control group of subjects with healthy eyelid margins, to investigate the potential value of cross-polarized light technology in blepharitis assessment. Additionally, since the diagnosis of blepharitis was not standardized (19), we not only diagnosed patients by experienced specialists of ocular surface disease, but also collected samples of eyelid secretions from all subjects in this study. By performing principal component analysis (PCA) on eyelid secretion samples, we aimed to validate the reliability of our patient selection criteria, laying a foundation to explore correlations of redness indices with clinical signs and secreting proteins.

## Materials and methods

### Subject

In this study, 12 adult Asian patients with recurrent blepharitis were enrolled in the case group. All patients were diagnosed with mixed blepharitis and had a medical history of at least 6 months. A total of 24 healthy controls, matched for age and gender in a 1:2 ratio to the case group, were recruited as the control group. Population characteristics (age, gender) and medical histories (including chalazion, hordeolum, and eyelid surgery) of both groups were documented. The upper eyelids of the right eyes in both two groups (or the eye with more severe blepharitis in the case group) were assessed. The privacy rights of human subjects have been observed and informed consent was obtained from subjects, and all potential effects were fully explained. This study was approved by the Institutional Review Board of Eye & ENT Hospital, Fudan University, and adhered to the tenets of the Declaration of Helsinki.

Diagnosis of blepharitis was made by two independent specialists in ocular surface diseases. The diagnostic criteria referenced the AAO Blepharitis Preferred Practice Pattern (20) and the Canadian Consensus (21). The criteria included: (1) clinical signs including debris, telangiectasia, swelling, hyperemia of the eyelid margin, crusting of lashes and thickened meibum; (2) presence of common blepharitis symptoms for more than 6 months, such as burning sensation, irritation, and redness; and (3) recurrent blepharitis relapses of at least three times. The exclusion criteria were as follows: (1) previous ocular surgery or trauma within the past 6 months; (2) upper eyelid defect; (3) acute viral or parasitic infection of the eyelid skin. These exclusion criteria also applied to the control group.

### Assessments

#### Ocular–surface indices

(1) Tear meniscus height (TMH): TMH is defined as the perpendicular length from the middle of the inferior tear meniscus to the lower eyelid margin, measured by the noncontact ocular analyzer Keratograph 5M (OCULUS, Wetzlar, Germany). (2) Tear break–up time (TBUT) and cornea staining: Following fluorescein instillation, TBUT was measured three consecutive times, and the median value was recorded for each patient. Cornea staining was assessed in five areas (optical–diameter, nasal, temporal, superior, and inferior) after fluorescein application. Each area was scored for superficial punctate keratopathy on a scale of 0 to 3, and the total scores from all five areas were summed.

#### Meibomian gland (MG) indices

1) Meibum quality: The quality of meibum from eight glands in the nasal and middle parts of the upper eyelid meibomian glands (MG) was evaluated on a scale of 0–3: 0 = clear, 1 = cloudy, 2 = cloudy with debris (granular), and 3 = thick, toothpaste-like. The total scores for these eight glands were summed. 2) Expressibility: The expressibility of five nasal MGs in the upper eyelid was assessed on a scale of 0–3 per gland: 0 = all glands expressible, 1 = 3–4 glands expressible, 2 = 1–2 glands expressible, and 3 = no glands expressible. 3) MG dropout: MG morphology was observed through the infrared images of everted upper eyelids via Keratograph 5M (OCULUS, Wetzlar, Germany). The “string-like” structures were defined as MGs. The percentage of lost MG structures was defined as meibomian gland dropout, which was calculated by Image J software (National Institutes of Health, Bethesda, MD, USA).

#### Proteomics

In this study, we utilized data independent acquisition (DIA) technology for proteomic analysis. Samples of eyelid margin secretions were collected from the required eye of all subjects. After protease digestion of the extracted proteins, liquid chromatography-mass spectrometry (LC-MS) was employed to analyze the peptide fragments. Relative quantitation of the proteins corresponding to these peptide fragments was achieved by comparing the signal intensities of corresponding peptide fragments in different samples, and quantitative analysis of the relative expression levels of proteins in the two different sample groups was them conducted. Additionally, principal component analysis (PCA) and Gene Ontology (GO) enrichment analysis of differential proteins were performed based on the expression levels of reliable proteins.

#### Redness area proportion

**FIGURE 1:**
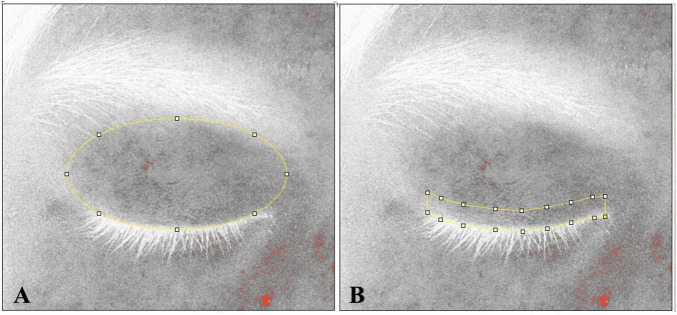
The measurement range for eyelid and eyelid margin. The measurement range is indicated by yellow lines. **(A)** The measured area of eyelid extends from eyebrow to eyelashes, following the natural shape of the human eyelid. **(B)** The measured range of the eyelid margin was specified as within 3 mm above the eyelashes.

To assess eyelid redness by cross-polarized light, we used Vplus^®^ (Fuhuan Science and Technology Ltd., Shanghai, China) in this study. Vplus^®^ is a professional skin image capture and analysis instrument based on a series of controlled multi-channel light environment, which includes the cross-polarized light. Under operator guidance, subjects placed their chin on a chin rest integrated into the Vplus^®^ instrument. They were required to maintain a stable head position, with relaxed facial expressions and gently closed eyes. The device then automatically collected high-definition images. Researchers further processed the captured signals of abnormally increased telangiectasis and hyperemia in eyelid skin, using ImageJ software. The superior palpebral arch of arteries spans the length of the upper eyelid, positioned approximately 2–3 mm above the eyelid margin (22). Therefore, two detection areas were set: (1) the overall eyelid, roughly elliptical in shape, extending from the eyebrow superiorly to the lid margin inferiorly; and (2) a 3 mm-height zone along the eyelid margin (Figure 1), approximately arcuate in shape. These two areas spanned the entire palpebral fissure from medial to lateral canthus. Researchers calculated the area proportion of increased redness signals within these two areas, termed as the redness area proportion of the eyelid (RAE%) and the redness area proportion of the eyelid margin (RAM%), respectively.

### Statistical methods

Data were analyzed using SPSS R27.0.10 (IBM Corp, America). Nonparametric tests (Wilcoxon test) were conducted for variable analysis. Linear Pearson correlation coefficients were calculated for normally distributed values, while linear correlation coefficients (Spearman test) were calculated for non-normally distributed values. The statistical significance level was set at 0.05.

## Results

### Population characteristics and clinical indices

**FIGURE 2:**
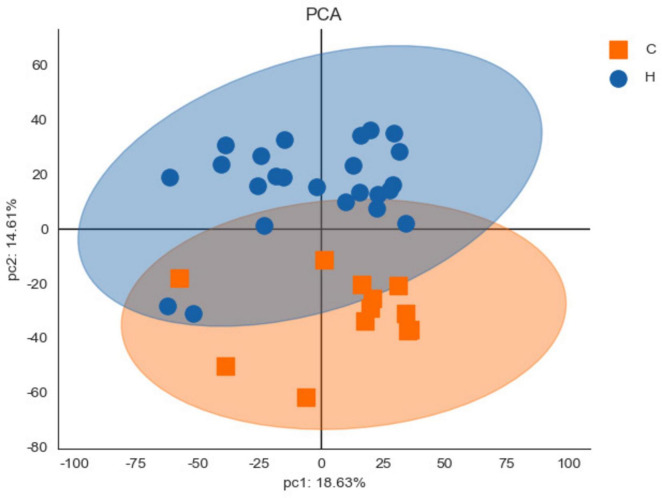
The principal component analysis (PCA) score chart. The horizontal axis (pc1) represents the interpretation rate of the first principal component, and the vertical axis (pc2) represents the interpretation rate of the second principal component. Each point on the chart represents a sample, allowing visual distinction between samples. Significant differences between the case group (C) and the health group (H) were identified.

In Table 1, the population characteristics, and clinical indices of the two patient groups adhered to the study design, with age and gender 1:2 matched. The case group included 12 patients, with a mean age ± standard deviation (SD) of 30.83 ± 8.85 years, 6 females and 6 males. The health group consisted of 24 patients, with a mean age of 30.83 ± 8.85 years and a gender distribution of 12 females and 12 males. Regarding past medical history, a significantly higher proportion of subjects in the case group (83.33%) had a history of chalazion/hordeolum compared to the health group (25.00%, *P* = 0.004). No statistically difference was observed between the two groups in terms of the proportion of subjects who had undergone eyelid surgery (*P* = 0.327). Additionally, aside from TMH, the case group exhibited poorer ocular-surface indices (including TBUT and cornea staining) and meibomian gland (MG) indices (including meibum quality, MG expressibility, and MG dropout) compared to the health group with statistically significance (all *P* < 0.05). Those results coordinated with the diagnosis of blepharitis in the case group. Figure 2 presents the PCA score chart, which demonstrated significant differences in protein expression of eyelid secretions between the case group and the health group. The PCA results provided further evidence for the reliability of distinguishing blepharitis patients from healthy controls in this study.

**TABLE 1:** Characteristics and clinical indices of the population.

Variables	Case group	Health group	*P*-value
	*n* = 12	*n* = 24	
Age (mean ± SD, year)	30.83 ± 8.85	32.13 ± 6.38	0.655
Gender (female/male, *n*)	6/6	12/12	1.000
Chalazion/hordeolum (%)	83.33	25.00	0.004*
Eyelid surgery (%)	33.33	12.50	0.327
TBUT (mean ± SD, s)	2.00 ± 1.48	8.33 ± 4.15	<0.001*
TMH (mean ± SD, mm)	0.27 ± 0.07	0.24 ± 0.07	0.280
Cornea staining (mean ± SD)	3.08 ± 5.05	0.00 ± 0.00	0.045*
Meibum quality (mean ± SD)	5.75 ± 5.08	0.04 ± 0.20	<0.001*
MG expressibility (0/1/2/3, *n*)	4/3/4/1	22/2/0/0	0.002*
MG dropout (mean ± SD, %)	41.18 ± 15.45	18.25 ± 7.27	<0.001*

SD, standard deviation; BKC, blepharokeratoconjunctiviti; TBUT, tear break-up time; TMH, tear meniscus height; MG, meibomian gland.

*p < 0.05, considered statistically significant.

### Redness area proportion

**FIGURE 3:**
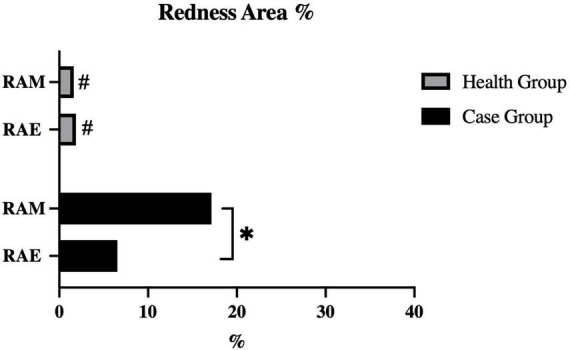
Quantification of redness area ratio at different eyelid areas. RAE, redness area ratio of eyelid; RAE, redness area ratio of margin. #*P*-value < 0.05 for intergroup compared between case group and health group. *P-value < 0.05 for intragroup compared between eyelid and margin.

**FIGURE 4:**
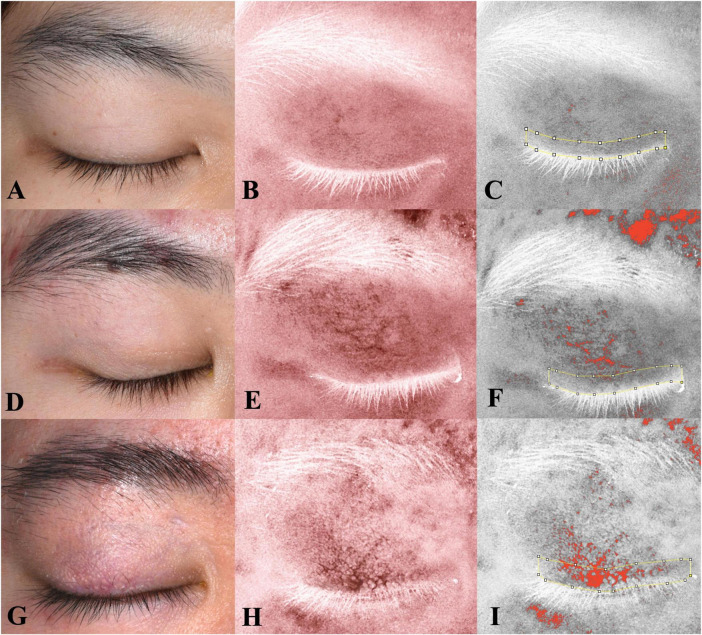
Eyelid images under different lighting and processing conditions with fixed distance and angle. The left row shows images under ordinary white light; the middle row, under crossed-polarized light (clearly showing telangiectasis and hyperemia after processing); and the right row, also under crossed-polarized light with further processing to extract hemoglobin signals. The measurement range of the eyelid margin is indicated by yellow lines. **(A–F)** The images of two subjects in the health group; **(G–I)** the images of one patient with blepharitis in the case group. Consistent with Figure 3, the patient with blepharitis **(G–I)** exhibited concentrated signals on eyelid margin, with a significantly higher redness area ratio of the eyelid margin (RAM) compared to other eyelid area. This characteristic phenomenon was absent in subjects with healthy margins **(A–F)**, even in those with poor skin condition **(D–F)**.

Figure 3 shows the characteristics of the redness area proportion among two groups. The mean ± SD values of the redness area proportion for the eyelid (RAE%) and eyelid margin (RAM%) in the health group were 1.88 ± 2.53% and 1.63 ± 2.04%, respectively. Among healthy subjects, both regions exhibited low proportions, without statistically difference between them (*P* = 0.548). In contrast, patients with recurrent blepharitis showed distinct findings. Specifically, the case group had the RAE% of 6.54 ± 7.20% (mean ± SD) and the RAM% of 17.14 ± 18.90% (mean ± SD), which were both significantly higher than the corresponding values in the health group (all *P* < 0.05). Additionally, within the case group, RAM% was significantly higher than RAE% (*P* = 0.019). In other words, the redness in recurrent blepharitis patients was predominantly concentrated within the area of eyelid margin rather than the whole eyelid. In Figure 4, this characteristic manifestation was more clearly demonstrated in the photos of specific cases. Figures 4G–I showed that telangiectasis and hyperemia in the case group were primarily localized around the superior palpebral arch. This phenomenon was absent in Figures 4A–F, which showed healthy eyelid margins in health group. Figures 4A–C were taken from a healthy control without special abnormalities in eyelid margin or skin, and no obvious increase in redness was observed in the whole eyelid or eyelid margin. Figures 4D–F were collected from a healthy control with acne on the skin, and redness signals were visible on the periorbital skin and the superior part of upper eyelid. However, the redness of eyelid margin in Figure 4F was at a similar level comparable to that in Figure 4C. Furthermore, in Figure 4, the vertical rows of photos revealed that with the help of cross-polarized light (Figures 4B, E, H), especially after abnormal redness signals were processed (Figures 4C, F, I), the changes were more pronounced than those under ordinary white light (Figures 4A, D, G). This comparison under different lighting and processing conditions suggested the application value of cross-polarized light among patients with blepharitis.

### Correlations with related indices

The researchers analyzed the correlations between the redness area proportion and related indices among a total of 36 subjects. In Table 2, neither RAE% nor RAM% had statistically correlations with age, gender, or past medical history (including chalazion/hordeolum and eyelid surgery) (all *P* ≥ 0.05). Additionally, both RAE% and RAM% had positive correlations with clinical indices of adjacent tissues statistically (all *P* < 0.05). Except for MG expressibility, RAM% showed stronger correlations than RAE% with cornea staining (RAE%, *r* = 0.521, *P* < 0.001; RAM%, *r* = 0.584, *P* < 0.001), meibum quality (RAE%, *r* = 0.381, *P* = 0.022; RAM%, *r* = 0.383, *P* = 0.021), and MG dropout (RAE%, *r* = 0.347, *P* = 0.038; RAM%, *r* = 0.348, *P* = 0.037). Finally, outside of the table, two tear film indices, TBUT and TMH, did not show statistically correlations with redness area proportions (all *P* ≥ 0.05). It suggested that RAM% possibly had a closer relationship with other clinical indices compared to RAE%.

**TABLE 2:** Correlation between redness indices and other related indices (*n* = 36).

Characteristic variables		Age	Gender	Chalazion/hordeolum	Eyelid surgery
RAE%	r	−0.142	−0.073	0.315	0.125
	*P*-value (two-tailed)	0.410	0.673	0.061	0.468
RAM%	r	−0.198	−0.114	0.291	0.287
	*P*-value (two-tailed)	0.247	0.508	0.086	0.089
**Clinical variables**		**Cornea staining**	**Meibum quality**	**MG expressibility**	**MG dropout**
RAE%	r	0.521*	0.381*	0.585*	0.347*
	*P*-value (two-tailed)	<0.001	0.022	<0.001	0.038
RAM%	r	0.584*	0.383*	0.497*	0.348*
	*P*-value (two-tailed)	<0.001	0.021	<0.001	0.037

RAE, redness area ratio of eyelid; RAE, redness area ratio of margin; MG, meibomian gland.

*P < 0.05.

**FIGURE 5:**
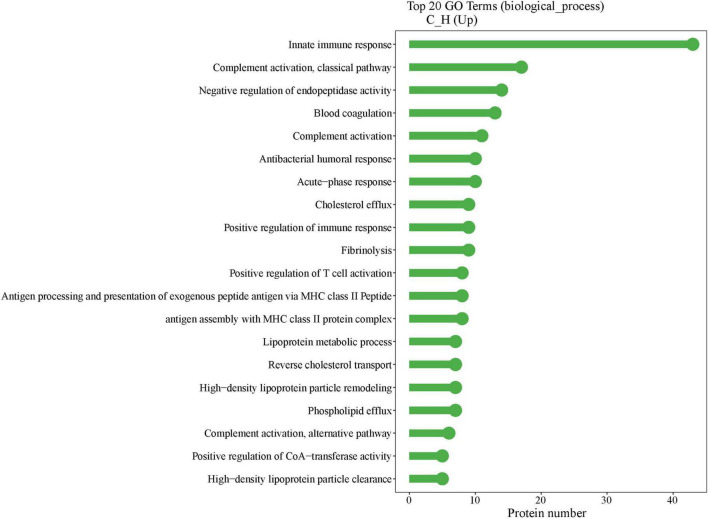
Gene ontology (GO) enrichment results of biological process. The *x*-axis indicates the number of proteins in the corresponding terms, and the y-axis lists the GO terms. The highest number of GO-enriched proteins was found in innate immunity.

**FIGURE 6:**
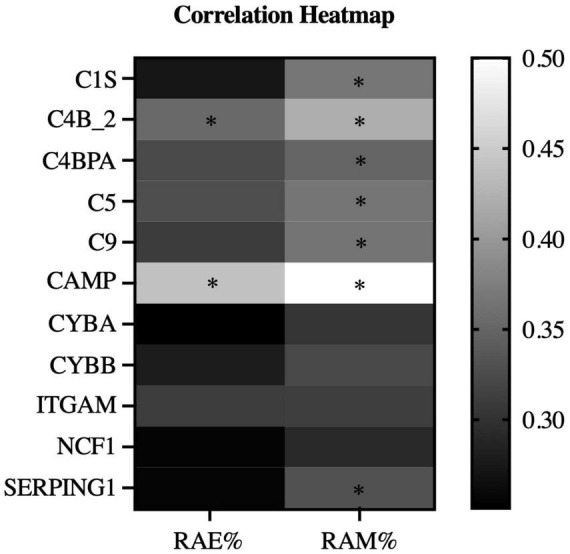
Correlation heatmap of redness indices and protein expression level (*n* = 36). The heatmap showed the correlation between the redness area ratio and the relative expression level of protein in all subjects. The color depth of the heatmap distinguishes the correlation coefficient values. RAE, redness area ratio of eyelid; RAE, redness area ratio of margin. *P-value < 0.05.

Figure 5 displays the GO enrichment results for the biological processes among all subjects (*n* = 36). The top two enriched terms were “innate immune response” and “complement activation, classical pathway.” Researchers selected representative proteins within these two terms, and further analyzed the correlations between the redness area proportion and the relative protein expression levels among all subjects. The heatmap presented in Figure 6 clearly shows that RAM% (C1S, C4B_2, C4BPA, C5, C9, CAMP, SERPING1, all *P* < 0.05) was positively associated with more proteins and had higher positive correlation coefficients compared to RAE% (C4B_2, CAMP, all *P* < 0.05). Therefore, compared to RAE%, RAM% possibly had a closer relationship with the differential protein expression of eyelid margin secretion.

## Discussion

Blepharitis is a common and complex disease. The consensus among ophthalmologists regarding blepharitis is, paradoxically, the lack of consensus on this disease (5, 19). This is primarily manifested in 1) the absence of standardized diagnostic criteria and quantifiable assessment method (14), and 2) the unclear pathogenesis in patients with recurrent relapses (7). These issues mutually hinder each other, significantly limiting the progress in this field and impairing the comparability among existing studies. For instance, there is a scarcity of prevalence reports, with a wide range from 12 to 47% (1, 2), and one of these few studies even relied on random-digit dialing for data collection (1). Therefore, we are trying to establish an objective and quantifiable evaluation method for blepharitis, particularly for the recurrent blepharitis that greatly troubled patients. This study aims to increase the detection rate of blepharitis and the comparative value among studies.

The selection of the starting point for blepharitis evaluation warrants careful consideration. A lot of previous studies have focused on the etiology of blepharitis, such as bacterial culture and demodex positivity (5, 23). However, the pathogenic roles of bacteria and demodex mites in blepharitis are still controversial (1, 4). Bacterial culture positivity was reported as 39.4% in patients with blepharitis and 33.3% in healthy controls (23), indicating a minimal difference between patients and healthy subjects. Additionally, researchers have continuously reported that immune-induced inflammatory responses may also play a role in the mechanism of blepharitis (2, 24, 25). Therefore, inflammation remains the core part of blepharitis, and we believe that redness, a characteristic sign of inflammation (including telangiectasis and hyperemia), is highly suitable for evaluation.

Meanwhile, dermatologists have already had a mature observation technique for chronic inflammatory diseases, known as cross-polarized light (15–17). Compared to visual inspection, cross-polarized light can more prominently and clearly present abnormally increased redness signals (Figure 4). Dermatologists have also reported observing telangiectasis in the eyelid area of patients with rosacea, which is closely related to blepharitis. However, due to a lack of understanding on ocular anatomy and function, the examed zones described in that report did not fit eyelid and eyelid margin well (26). In this case-control study, we defined the area according to the specific blood supply of eyelid margin, superior palpebral arch. Interestingly, we found that patients with blepharitis had characteristic changes in redness: abnormally increased redness signals were significantly concentrated around palpebral arch (RAM% > RAE%). This was not observed in healthy controls. And even in some healthy subjects with abnormal condition of facial skin, the redness of their eyelid margins was similar to other healthy controls. These results suggested the application value of using cross-polarized light in evaluating patients with blepharitis.

Furthermore, in further correlation analysis, we found that RAM% had higher correlations with clinical indices than RAE% (Table 2), including cornea staining, meibum, and MG dropout. This was consistent with the characteristic image of redness in patients with recurrent blepharitis. These indices evaluated tissues directly related to the eyelid. Tear film indices and MG expressibility were influenced by other factors too, which potentially explained their different correlations with redness area proportions. Additionally, age, gender, and eyelid-related medical history had minimal impact on redness area proportions. Protein analysis of eyelid margin secretions from both groups provided additional supportive information. Firstly, the distinct distribution of the health group and case group in the PCA chart (Figure 2) supported the reliability of subject enrollment. Secondly, after GO enrichment analysis in the biological process, we found that the proteins with different expression levels (Figure 5) between two groups were mainly enriched in these two terms “innate immune response” and “complement activation, classical pathway.” Complement activation is part of the innate immune system (27), and both terms are associated with tissue inflammation. For example, complement C5 can mediate local inflammatory responses and cause vasodilation and increased capillary permeability through chemotactic activity (11–13). Our analysis revealed that RAM% was positively associated with more relevant proteins (such as C5) compared to RAE%. Among the proteins had statistical correlations with both RAM% and RAE%, RAM% had higher positive correlation coefficients with these proteins. Therefore, RAM% is suggested as a more precise and suitable index than RAE%, for reflecting the clinical symptoms and inflammatory status of patients with blepharitis.

Of course, this study has some inevitable limitations. Firstly, due to the lack of a gold standard for blepharitis diagnosis, the researchers could not directly obtain a cutoff value for RAM%. Secondly, to increase its application value, it is necessary to expand the sample size to obtain more reliable and stable RAM% ranges for both blepharitis and healthy populations in the future. Other limitations of this study point to the direction of optimizing the methods and instruments for ocular use. We will try to resolve the problem of eyelash obstruction above lower eyelids and explore the potential application in the mucosal areas. Improving image acquisition protocol and specialized algorithm maybe be helpful to subtracting eyelash artifacts from the images. In summary, using cross-polarized light to examine the eyelid can quickly, conveniently, and non-invasively observe characteristic changes in patients with recurrent blepharitis. And the index, RAM%, can standardize and quantify the inflammatory status of blepharitis. Obtaining RAM% through cross-polarized light has great potential value for the diagnosis and severity assessment of blepharitis.

## Data Availability

The datasets presented in this article are not readily available because due to the protection of the privacy of the data content, the database cannot be disclosed. Requests to access the datasets should be directed to YY, yinyueyinyue@126.com.
